# Adjusting the Morphology and Properties of SiC Nanowires by Catalyst Control

**DOI:** 10.3390/ma13225179

**Published:** 2020-11-17

**Authors:** Chuchu Guo, Laifei Cheng, Fang Ye, Qing Zhang

**Affiliations:** Science and Technology on Thermostructural Composite Materials Laboratory, Northwestern Polytechnical University, West Youyi Rd, No. 127, Xi’an 710072, China; ccguo@mail.nwpu.edu.cn (C.G.); chenglf@nwpu.edu.cn (L.C.); yefang511@nwou.edu.cn (F.Y.)

**Keywords:** silicon carbide nanowires, vapor-solid-liquid mechanism, oxide-assisted growth mechanism, photoluminescence, thermal stability

## Abstract

We report on the growth of SiC nanowires on a single crystal Si substrate by pyrolysis of polycarbosilane and using two catalyst (Al_2_O_3_ and Ni) films with different thickness (2, 4, and 6 nm). The catalyst films were deposited on the Si substrate, and the SiC nanowires were grown according to two mechanisms, i.e., the oxide-assisted growth mechanism and vapor- liquid-solid mechanism. As a result, pearl-chain-like SiC nanowires and straight SiC nanowires were obtained. The prepared nanowires exhibited excellent photoluminescence properties, emission spectra displaying two emission peaks at 395 and 465 nm, and have good thermal stability below 1000 °C. The experimental results revealed the importance of the catalyst in controlling the morphology and properties of SiC nanowires.

## 1. Introduction

In the nanometer scale, the surface effect, small size effect, quantum size effect and macroscopic tunneling effect dramatically change the physical and chemical properties of materials [1,2,3,4]. For instance, SiC nanowires, a non-oxide ceramic material, attracted great interest as a highly promising nanomaterial for many industrial applications due to their superior electric and mechanical properties, as well as heat, corrosion, and high temperature oxidation resistance [5,6,7]. These unique properties enable SiC nanowires to be used as ideal candidates for nanodevices for sensing and biosensing applications, or in composite materials as a reinforcement [8,9,10]. To the key role, SiC nanowires are widely used in aerospace, nuclear, braking systems and other industrial fields [11,12,13]. The development of high-performance materials and the maturity of preparation technology have stimulated researchers to design and create the SiC nanowires with higher performance, which further promoted deeper research on the growth mechanism of SiC nanowires.

Oxides are commonly used as catalysts to grow nanowires through the oxide-assisted growth (OAG) mechanism, whereas transition metals are often used as catalysts for the growth of nanomaterials by the vapor-liquid-solid (VLS) mechanism. Zhang [14] studied the growth of Si nanowires with uniform size and long length by OAG and VLS mechanisms, aiming at achieving a controlled growth of nanowires. Kang [15] used Au catalyst to prepare III-V semiconductor nanowires with a certain angle and extended (111) direction on the (001) Si substrate by the VLS mechanism. Zhang [16] prepared bamboo-like SiC nanowires by doping Al catalyst during the pyrolysis of polycarbosilane (PCS). The findings highlighted the positive role played by the catalyst during the growth of semiconductor and oxide nanowires. Wang [17] reported the synthesis of a Si nanowire structure, using tin particles as a catalyst, and by adjusting the periodic volume change of the catalyst, liquid tin segments periodically appeared in the Si nanowires during the growth process. The results clearly show that this abnormal growth is controlled by the dual effects of VLS and OAG. In the process of preparing Si nanowires, M. Agati [18] discovered that the nanowires are grown catalyzed by a silicon core (with a diameter of 2 nanometers) and a silicon shell. The combination of advanced transmission electron microscopy technology proved that the growth of long Si nanowires is carried out by the OAG mechanism, while the growth of shorter Si nanowires is carried out by the VLS mechanism. Wang [19] uses the electron beam evaporation method under ultra-high pressure to prepare one-dimensional silicon nanowires. The results show that during the VLS growth process, Si nanowires can be formed on the Si surface, but Si nanowires cannot be formed on the SiO_2_ surface. But in the OAG process, Si nanowires can grow on the surface of SiO_2_. Nevertheless, only a few reports on the growth of SiC nanowires in the presence of catalysts and different growth mechanisms are available in the literature.

This work is focused on the preparation of SiC nanowires, displaying different morphologies, by changing the type and content of catalysts loaded on the substrate. The nanowires were grown on the single crystal silicon substrate by VLS and OAG mechanisms. The phase composition, microstructure, and morphology, as well as the photoluminescence properties and thermal stability of the prepared SiC nanowires were analyzed. The growth mechanisms of SiC nanowires were described in detail, providing fundamental knowledge for the rational control of the morphology of SiC nanowires.

## 2. Experimental

### 2.1. Synthesis of Samples

The 30 wt.% PCS precursor (Xiamen University) was mixed with xylene under magnetic stirring for 30 min. A multifunctional ion beam assisted deposition (IBAD) device is adopted. The pressure during the deposition process: 2.5 × 10^−2^ Pa~3.2 × 10^−2^ Pa, the ion beam energy is 600 eV, and the deposition rate is 0.05 nm/s. The deposition time is controlled to obtain nano-films of different thicknesses (2 nm, 4 nm and 6 nm), and the preparation of the catalyst film is characterized by the weight gain and surface morphology of the substrate. The main components of the target (wt.%) are: 99.99% Ni and Al_2_O_3_. The basic material is a single crystal Si (111) wafer, and the substrate temperature is lower than 100 °C during the deposition process. The precursor was then poured into a crucible, which was transferred to a tube furnace. The substrate with catalyst film was placed downstream 5 cm from the precursor. The air in the furnace was replaced with Ar gas at a flow rate of 40 SCCM (standard cubic centimeter per minute). The temperature was raised to 1350 at a rate of 5 °C/min and held for 3 h. The obtained products were named as A1, A2, A3, respectively for the samples prepared with Al_2_O_3_ catalyst film at a thickness of 2, 4, and 6 nm, and N1, N2, N3, respectively for the samples prepared with Ni catalyst film at a thickness of 2, 4, and 6 nm.

### 2.2. Characterization of Samples

The obtained SiC nanowires were coated with a 5 nm layer of gold for scanning electron microscopy (SEM) imaging. The images were taken using a field emission scanning electron microscope (FE-SEM, S-4700, Tokyo, Japan). Samples were also observed by transmission electron microscopy (TEM) using a FEI Talos F200X microscope (Hillsboro, OR, USA). Crystal phases were characterized by an X-ray diffractometer (X-ray diffraction, XRD, AXS D8, Karlsruhe, Germany) via grazing incidence mode with an incidence angle of 2°, and MDI Jade software (California City, CA, USA) was used for fitting calculation of crystallization rate and crystallite size as an auxiliary method to analyze the evolution of sample structure and phase composition by using the following equations:

(1) $$\mathrm{Crystallinit}=\frac{\mathrm{diffraction}\;\mathrm{peak}\;\mathrm{strength}}{\mathrm{total}\;\mathrm{strength}}\times 100\%$$

(2) $$\mathrm{Crystallite}\;\mathrm{size}=\frac{K\lambda }{\mathrm{FW}\left(S\right)\cos\theta }$$

K is a constant, λ is the wavelength of an X-ray, FW(S) is the width of the sample, and *θ* is the diffraction Angle.

The photoluminescence properties were characterized by X-ray fluorescence spectrometry (Axios-X, Almelo, Netherlands), and the thermal stability of the samples was measured with thermogravimetric analysis-differential scanning calorimetry (TG-DSC) equipment at 10 °C/min to 1400 °C air atmosphere (GCMS QP2010 PLUS, Waltham, MA, USA).

## 3. Results and Discussion

**Figure 1:**
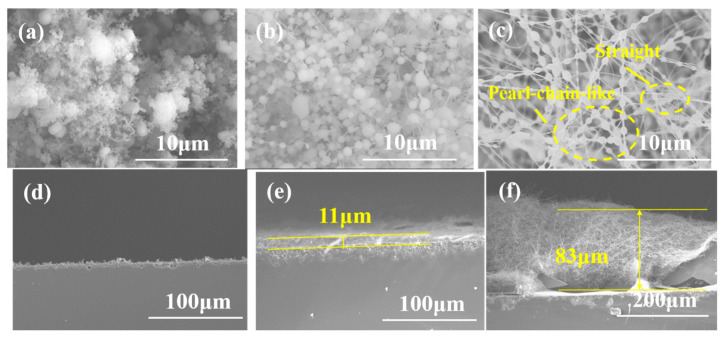
Surface morphology scanning electron microscopy (SEM) images of (**a**) A1, (**b**) A2, (**c**) A3 SiC nanowires, Cross-section morphology SEM images of (**d**) A1, (**e**) A2, (**f**) A3 SiC nanowires.

**Figure 2:**
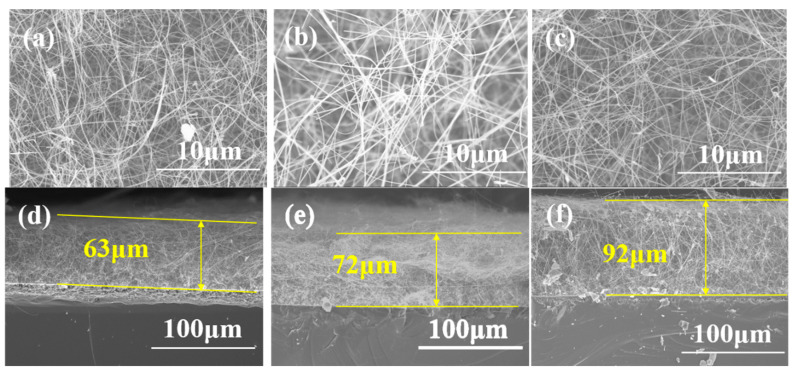
Surface morphology SEM images of (**a**) N1, (**b**) N2, (**c**) N3 SiC nanowires, Cross-section morphology SEM images of (**d**) N1, (**e**) N2, (**f**) N3 SiC nanowires.

The morphology and growth mechanism of SiC nanowires were controlled by using different catalysts. The corresponding surface and cross-section morphology SEM images of all six samples are illustrated in Figure 1 and Figure 2.

As shown in Figure 1a, when the thickness of the Al_2_O_3_ catalyst is 2 nm, a large number of pearl-like beads are formed on the surface of the substrate, which are in intimate contact with the surface (Figure 1d). As the thickness of the catalyst film increases, SiC nanowires begin to form, but they are straight and co-exist with the pearl-chain-like nanowires (Figure 1b,c). Also, the thickness of the nanowire on the substrate surface becomes larger which obviously increased from 11 to 85 μm for the samples prepared with the Al_2_O_3_ film thickness of 4 and 6 nm, respectively (Figure 1e,f). The diameter of the straight nanowire is about 50 nm. The minimum diameter of the pearl-chain-like nanowire is about 100 nm, and the maximum diameter of the pearls is about 850 nm. The SEM images of the samples prepared with Ni catalyst are illustrated in Figure 2. As observed, the diameter of the SiC nanowires catalyzed by Ni film is about 50 nm while their surface is smooth and clean (Figure 2a–c). The cross-section image (Figure 2d) shows a mats-like arrangement of the nanowires on the substrate. As the thickness of the Ni catalyst increases to 4 and 6 nm, the thickness of nanowires increases from 63 to 92 μm, respectively, while the layer of nanowires is denser (Figure 2e,f). These results indicate that different growth mechanisms of the nanowires in the presence of the two catalysts. For A1, A2, and A3 samples, amorphous shells were observed on the surface of nanowires. This is a typical feature of the OAG growth mechanism, so it can be concluded that nanowires grow in accordance with the OAG growth mechanism [20,21,22]. In this case, the growth of SiC nanowires is assisted by semi-liquid Al_2_O_3_ when an Si-O-Al amorphous layer is formed on the surface of the nanowires, preventing the lateral growth of the nanowires. By contrast, the growth mechanism of the nanowires in N1, N2, and N3 samples is the VLS mechanism. In this case, the catalyst droplets form at lower temperatures. The catalyst droplet can be used as a template to control the morphology of the SiC nanowire at the top of the nanowire. Secondly, the solid-liquid interface is formed at the top of the nanowires, so that the reactants continue to crystallize at the interface to form nanowires.

**Figure 3:**
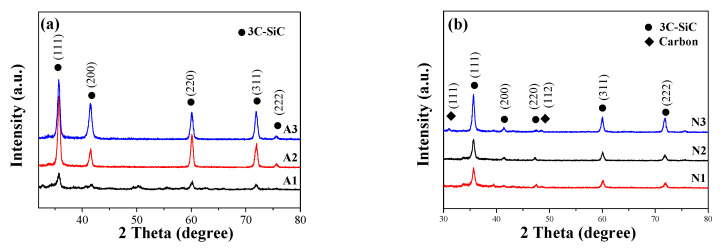
X-ray diffraction (XRD) patterns of SiC nanowires prepared with (**a**) Al_2_O_3_, (**b**) Ni catalysts.

Figure 3 displays the XRD patterns of SiC nanowires grown in the presence of the two different catalysts.

As illustrated in Figure 3a, all three samples showed typical diffraction peaks for 3C-SiC. When the thickness of Al_2_O_3_ is 2 nm, the characteristic peak strength of SiC is extremely weak. As the thickness of the Al_2_O_3_ catalyst film increases, the characteristic peaks of SiC gradually become sharp and clearly visible. This indicates that the catalyst increases, the amount of nanowires is larger or the crystallinity gradually increases. In Figure 3b, the typical diffraction peaks of SiC are displayed even at the lower thickness of the Ni film. However, the peaks become sharper as the thickness of the Ni film increases. To note, for the N3 sample, traces of C can be observed in the XRD pattern. The comparison of the crystallinity of samples reveals that a crystallization degree of 22.3% is obtained for the N3 sample, which is made of crystallites of 14.3 nm, whereas a crystallinity degree of 35.6% is determined for the A3 sample, consisting of crystallites of 21.1 nm (Table 1). This result is consistent with the SEM result. The sample with Al_2_O_3_ as catalyst has a larger diameter, so the overall crystallinity is higher. This is because the semi-liquid catalyst in the OAG growth mechanism is only attached to the surface of SiC nanowires, and the limiting force on the diameter of nanowires is weaker than that on the tip of the nanowire droplets in the VLS mechanism.

**Figure 4:**
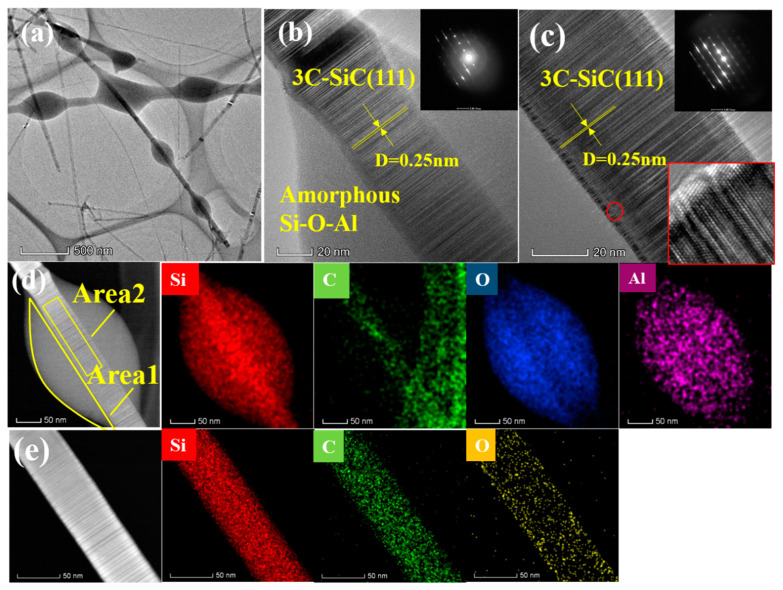
(**a**) Transmission electron microscope (TEM) image of A3 sample, (**b**) high-resolution transmission electron microscope (HRTEM) image and selected area electron diffraction (SAED) pattern of pearl-chain-like nanowire, (**c**) HRTEM image and SAED pattern of straight nanowire. (**d**) Energy-dispersive spectroscopy (EDS) mapping of pearl-chain-like nanowire; (**e**) EDS mapping of straight nanowire.

The microstructure and composition of the A3 sample are analyzed by high-resolution transmission electron microscopy (HRTEM), selected area electron diffraction (SAED), and energy-dispersive spectroscopy (EDS). The results are displayed in Figure 4.

Figure 4 shows representative HRTEM images of the A3 sample. It can be observed that two different morphologies co-exist in the sample, a bead-like morphology as well as a smooth and straight-shaped morphology (Figure 4a). The pearl-like beads of ~150 nm in diameter, which are placed alongside the nanowire, are amorphous. The core of the beads is crossed by a straight nanowire with a diameter of 50 nm. The *d*-spacing between two neighboring lattice fringes is 0.25 nm, according to Figure 4b. It can be observed from the SAED in the Figure 4b inset, that the crystal diffraction lattice co-exists with a halo, attributed to the amorphous phase. Hence, it can be stated that the core of the pearl-like beads is made of a SiC single crystal while the pearl-like structure is amorphous. From Figure 4c, it can be observed that the straight SiC nanowires, having a diameter of 50 nm, are grown in the (111) direction. In addition, defects such as twin defects can be observed inside the nanowires by magnifying high resolution images in red circles (Figure 4c). The EDS pattern of pearl-chain-like nanowire clearly shows that the amorphous layer mainly contains Si, O, and Al (Figure 4d). As listed in Table 2, in the Area 1, the amorphous Si–O ratio is close to 1:2 while a small amount of Al is also detected. The Si–C ratio of Area 2 is close to 1, and the atomic content of oxygen is 2.65%, indicating that the core of the nanowire is composed of 3C–SiC single crystals. The microstructure and composition of the smooth and straight nanowires in A3 are the same as the core of the beads discussed above (Figure 4e).

**Figure 5:**
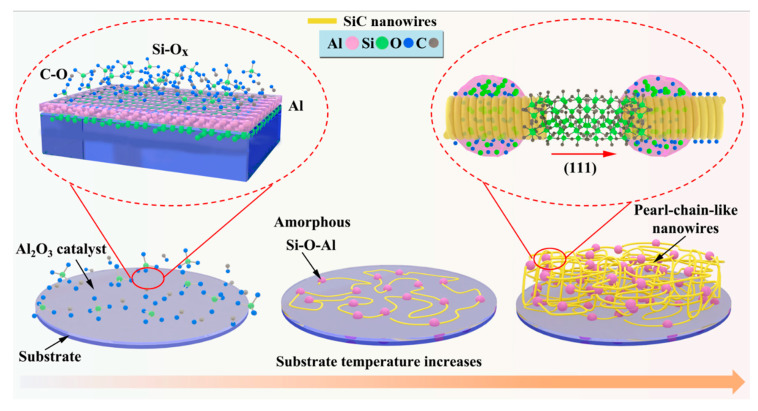
Schematic diagram of growth mechanisms of SiC nanowires loaded with Al_2_O_3_ catalysts.

Since no other metal elements participate in the reactions during the synthesis, but only Al_2_O_3_, it is evident that the nucleation and growth of the A1, A2, and A3 samples are governed by the OAG mechanism. Figure 5 schematically illustrates the growth of SiC nanowires in the presence of the Al_2_O_3_ catalyst via the OAG mechanism.

In the initial stage of the reaction, the Al_2_O_3_ film deposited on the Si substrate is strongly bonded to the substrate, which limits the agglomeration of the catalyst on the substrate surface. The pyrolysis temperature of 1350 °C is very close to the melting point of the silicon wafer, which weakens the bonding force between the Al_2_O_3_ thin layer and the silicon wafer. The released reactive atoms react with the pyrolysis byproduct gas (CO and SiO_X_) of the PCS, the available bonds are directed toward the surface, and the diffused Al atoms form a Si–O–Al amorphous layer. This amorphous layer plays the role of adsorbent of reactive gases and promotes the formation of SiC nanowires with a certain crystal orientation. During the growth of SiC nanowires, the oxygen and aluminum atoms in Si–O–Al may be expelled by silicon atoms, which diffuse to the edges of the crystal and form amorphous protective shell, which also guides the growth direction of the nanowires. Therefore, the atomic content of oxygen in the pearl-shaped amorphous layer is as high as 63.39%. Overall, the highly reactive Si–O–Al layer on the top of the SiC nanowire acts as a collector of gaseous Si–C, whereas the amorphous layer on the side of the nanowire prevents the increase of the nanowire diameter. Thus, a single crystal SiC nanowire with a straight center is formed. It is assumed that the twin defects of SiC nanowires are one of the driving forces of the growth along one direction. The existing twin dislocations in the growth direction and the formation of facets with low surface energy can also improve growth rate of nanowires along the (111) crystal plane. Twin steps are more likely to adsorb atoms. When the rate of amorphous adsorption in the reaction system is greater than the crystallization rate of SiC, the pearl-like amorphous clusters appear on the nanowires, forming the pearl-chain nanowires.

**Figure 6:**
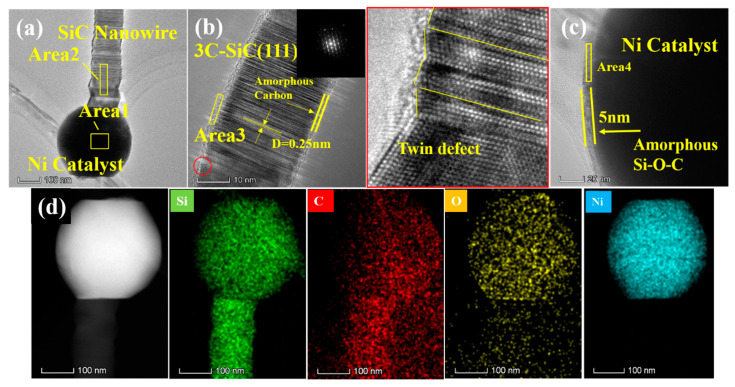
(**a**) TEM image of N3 sample; (**b**) HRTEM image and SAED pattern of nanowire; (**c**) HRTEM image of catalyst; (**d**) EDS mapping of N3 sample.

Representative HRTEM images of N3 sample are depicted in Figure 6.

As can be seen from Figure 6a, Ni catalyst is placed on the top of SiC nanowires, which is a typical feature of VLS mechanism [23,24,25]. This indicates that N1, N2 and N3 have grown according to the VLS mechanism [26,27]. The Ni catalyst controls the growth of SiC nanowires in the (111) direction. As shown, the average diameter of nanowires is 50 nm (Figure 6b). Many twin defects can be observed inside the nanowires, and a periodic sawtooth-shaped crystal structure of the SiC nanowires is noticed on the surface. As can be seen from the elemental analysis in Table 3, the catalyst droplet mainly contains 77.76% Ni and a small amount of Si, C and O on the surface. The elemental composition of single crystal nanowires is mainly Si and C with atomic ratios close to 1:1, and O with 2.38%. The amorphous layer coated on the surface of nanowires is mainly C. The SAED pattern shown in the illustration in Figure 6b shows a single crystal lattice. The high-resolution image magnified in red circles shows defects in nanowire growth, such as twin defects. The high-resolution image in Figure 6c shows that the diameter of the Ni catalyst droplet is about 200 nm. The surface of the droplet is covered by an amorphous layer of Si–O–C with a thickness of 5 nm. The EDS spectrum of N3 shows that the catalyst droplets on the top of the nanowires are composed of Si, C, O, and Ni, whereas the Si–O–C mainly exists on the surface of the catalyst droplet (Figure 6d).

**Figure 7:**
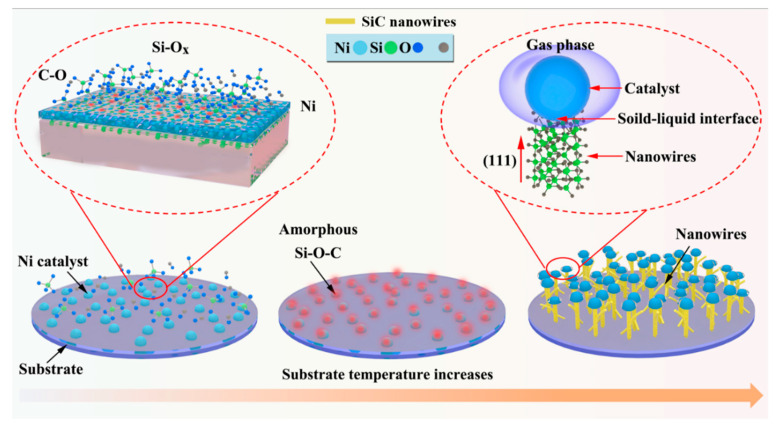
Schematic diagram of growth mechanisms of SiC nanowires loaded with Ni catalysts.

Figure 7 briefly illustrates the growth of SiC nanowires supported with Ni catalyst via the VLS mechanism.

As the increase of substrate temperature, Ni first forms small droplets on the substrate surface, followed by the formation of a liquid–solid interface. Subsequently, the by-product gas formed during PCS pyrolysis is continuously adsorbed on the liquid–solid interface and promotes the crystallization of Si–O–C amorphous phase. The extra unreacted C is coated on the surface of nanowires, resulting in the formation of SiC single crystal nanowires. In addition, the lowest surface-energy (111) growth is allowed. Under these conditions, the increase in nanowire diameter is limited and the single-crystal SiC nanowires with straight morphologies are produced. It is different from the bead chain-like nanowire prepared by using Al_2_O_3_ as the catalyst.

**Figure 8:**
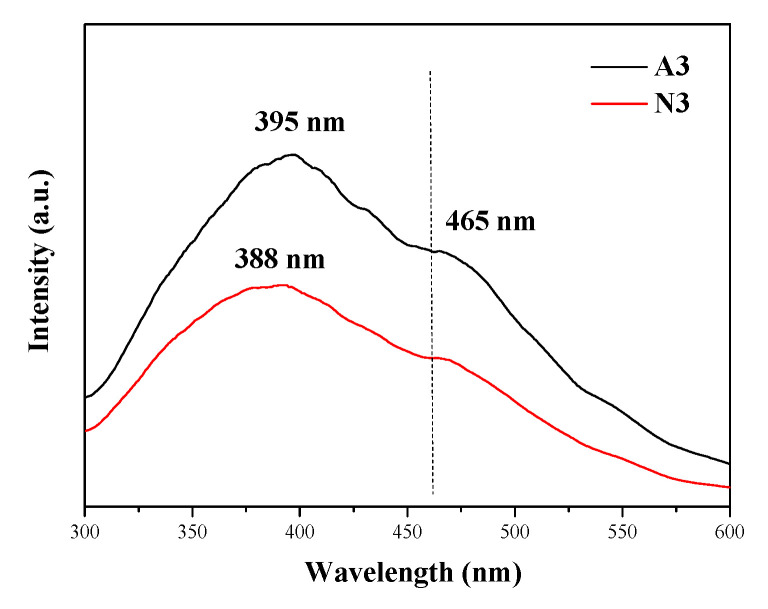
Photoluminescence spectra of A3 and N3 samples.

Figure 8 illustrates the photoluminescence spectra of A3 and N3 samples.

As shown in Figure 8, the photoluminescent spectra at 350 nm of A3 and N3 display two obvious emission peaks at ~395 and 465 nm, corresponding to 3.13 and 2.67 eV, respectively. This indicates that the prepared 3C-SiC nanowires emit within a wide range of wavelengths. However, the intensity of the emission peaks varies according to the catalyst type. It is obvious that the morphology and structural defects influence the photoluminescence characteristics of nano-SiC crystals [28]. Herein, the shape of the emission peaks is largely similar, and the center position of the emission peak changes minimally. However, compared to the conventional SiC crystal with a relatively larger crystallite size for which the emission occurs at 556 nm, a significant blue shift is noticed for both samples. At this point, the shape of the emission peak is basically similar, and the central position of the emission peak changes the least. However, the emission at 556 nm (2.39 eV) showed a significant blue shift in both samples when compared with conventional SiC crystals with a larger crystallite size. The emission peak of SiC nanowires at 465 nm has also been reported in other literature [29,30]. This can be explained by the quantum size effect, which is caused by the size constraint effect due to the small crystallite size and the displacement of emission peak due to the internal defect [31,32]. Compared with SiC crystals with larger crystallite size, single crystals with nanowire diameter of ~100 nm were prepared. As previously observed, there are complex accumulation defects in silicon carbide nanowires. The other peak value is concentrated at 400 nm, which is basically consistent with the values of 3C–SiC nanotubes [33] and SiC nanopores [32] The presence of a large number of amorphous phases in A3 leads to the formation of a bead chain with a diameter of 850 nm, and the presence of amorphous elements also leads to the blue shift of the emission peak center [34].

**Figure 9:**
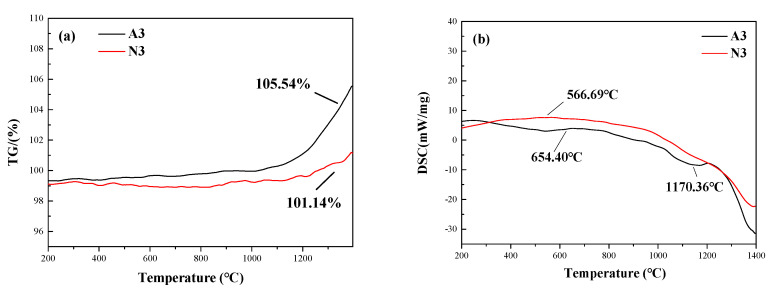
(**a**) Thermogravimetric (TG) and (**b**) differential scanning calorimetry (DSC) curves of A3 and N3 samples.

The TG and DSC curves of A3 and N3 are displayed in Figure 9.

The TG curves show no change before 1100 °C. However, above 1100 °C, a weight gain is observed for both samples, indicating that the SiC nanowire sample is further oxidized. The oxidation is more evident for the A3 sample, of which the residual mass is 105.54% (Figure 9a). In this case, the sample contains Al with a lower melting point (660 °C), while the content of amorphous Si–O–Al is high and easily oxidized, which is reflected by the weight gain. N3 samples showed a relatively low rate of mass change. This is because the excess carbon in the PCS precursor is consumed by the trace of oxygen, forming a thin amorphous layer of carbon on the surface of the nanowire [35]. It can be observed from the TEM image that there is an amorphous carbon layer on the surface of SiC nanowires, and the oxidation of the amorphous carbon layer will consume and result in weight loss. Meanwhile, the weight gain and mass cancellation of the amorphous crystallites and Si–O–C occur during the oxidation process. N3 shows absorption peaks at 566.69 °C, while the adsorption peak of A3 appears at 654.40 °C (Figure 9b). In addition, a peak appears at 1170.36 °C, likely due to the introduction of Al. Therefore, the thermal stability of the oxidizing substance has a great influence on the thermal stability of the nanowire at high temperatures.

## 4. Conclusions

1. SiC nanowires with pearl-chain-like morphology were prepared using Al_2_O_3_ as catalyst. Straight SiC nanowires were prepared with Ni as catalyst. The crystallinity of pearl-chain-like nanowires is slightly higher than that of straight nanowires. The results showed that the efficiency of the nanowires formation depends on the thickness of the catalyst film. Moreover, for the both SiC nanowire samples, the generation of twin structural defects was noticed.
2. The main mechanism governing the formation of SiC nanowires in the presence of Al_2_O_3_ is the OAG mechanism. In this case, the growth of nanowires is mainly controlled by the amorphous Si–O–Al coated on the surface of the nanowires while the pearl-like beads are generated due to the twin defects. The SiC nanowires grown in the presence of Ni catalyst follow the VLS mechanism. As the temperature increases, and in the presence of the catalyst, the crystallization of SiC is favored, generating the final nanowire structure.
3. SiC nanowire samples prepared with two different catalysts have emissions peaks in the visible range at 395 and 465 nm. The emission peaks blue shifted in comparison with the conventional micro-size SiC due to the nanosized dimension of the SiC nanowires, twin defects, and amorphous phases are identified in the samples.
4. The two samples have good thermal stability under 1000 °C in air atmosphere. Beyond this temperature, the mass of the material begins to change as a result of some slight oxidation of the material.

## Figures and Tables

**Table 1:** Crystallinity and crystallite size of SiC nanowires grown on the substrate surface in the presence of Ni or Al_2_O_3_ as catalysts.

Sample	Crystallinity/%	Crystallite Size/nm
A3	35.6	21.1
N3	22.3	14.3

**Table 2:** Elemental analysis results of different regions in A3 displayed in Figure 4d.

	Element	Si	C	O	Al
Atomic Fraction (%)					
Area 1		29.53	2.06	63.39	5.01
Area 2		52.81	44.54	2.65	–

**Table 3:** Elemental analysis of different regions in N3 corresponding to Figure 6a,b.

	Element	Si	C	O	Ni
Atomic Fraction (%)					
Area 1		12.91	7.79	1.54	77.76
Area 2		49.05	48.57	2.38	–
Area 3		11.68	79.43	8.89	–
Area 4		48.24	44.85	6.92	–
